# Successful treatment of *Mycobacterium ulcerans *osteomyelitis with minor surgical debridement and prolonged rifampicin and ciprofloxacin therapy: a case report

**DOI:** 10.1186/1752-1947-2-123

**Published:** 2008-04-27

**Authors:** Daniel P O'Brien, Eugene Athan, Andrew Hughes, Paul D Johnson

**Affiliations:** 1 Department of Infectious Diseases, The Geelong Hospital, Ryrie Street, Geelong, Australia; 2 Victorian Infectious Diseases Service (VIDS), Royal Melbourne Hospital, Grattan Street, Parkville, Melbourne, Australia; 3 Médecins Sans Frontières, Amsterdam, The Netherlands; 4 Department of Infectious Diseases, Austin Health and University of Melbourne, Bell Street, Heidelberg, Victoria, Australia

## Abstract

**Introduction:**

Treatment for osteomyelitis-complicating *Mycobacterium ulcerans *infection typically requires extensive surgery and even amputation, with no reported benefit from adjunctive antibiotics.

**Case presentation:**

We report a case of an 87-year-old woman with *M. ulcerans *osteomyelitis that resolved following limited surgical debridement and 6 months of therapy with rifampicin and ciprofloxacin.

**Conclusion:**

*M. ulcerans *osteomyelitis can be successfully treated with limited surgical debridement and adjunctive oral antibiotics.

## Introduction

*Mycobacterium ulcerans *is the third most common mycobacterial disease worldwide, occurring mainly in tropical regions. It usually causes a destructive skin and subcutaneous lesion, variously known as Buruli or Bairnsdale ulcer (BU). Up to 13% of cases in Africa have been reported to be complicated by osteomyelitis [1], however, this is rare in Australia. Osteomyelitis is usually treated by wide surgical excision, often resulting in significant morbidity. Recently there has been some evidence to support a beneficial role for antibiotics in the treatment of *M. ulcerans *disease [2], including our case series on the treatment of BU [3], but to the best of the authors' knowledge, there have been no other reports on their benefit in treating *M. ulcerans *osteomyelitis.

## Case presentation

We describe a case of *M. ulcerans *infection from temperate south-eastern Australia, involving six metachronous lesions and the eventual development of osteomyelitis. We believe this is the first reported case of *M. ulcerans *osteomyelitis that was cured with a combination of minor surgical debridement and prolonged antibiotic therapy.

A previously well 87-year-old, 55 kg woman presented with a painless ulcer on the right calf of 3 months duration. *M. ulcerans *was confirmed as the aetiological agent by histopathology testing, showing necrotising granulomatous inflammation with numerous acid-fast bacilli (AFB), a positive mycobacterial culture for *M. ulcerans *and a positive polymerase chain reaction (PCR) for *M. ulcerans *[4]. She was treated with wide surgical excision and primary closure, with clear histological margins.

Within 14 days, she developed a further *M. ulcerans *lesion on the left heel confirmed by suggestive histology and a positive PCR for *M. ulcerans*. This was treated with wide excision and a vascularised free flap, again with clear histological margins.

Despite adjunctive antibiotics (5 days of amikacin 900 mg daily, and ongoing rifampicin 450 mg daily, ethambutol 800 mg daily and clarithromycin 250 mg twice daily), a further lesion was found on her right buttock 25 days later. This was excised with clear histological margins. Although AFB were seen microscopically, and the tissue was PCR-positive for *M. ulcerans*, mycobacterial cultures were negative. At the same time, she suffered a severe febrile drug reaction with significant malaise and dehydration considered to be due to the clarithromycin. She thus commenced on 18 days of intravenous amikacin 900 mg daily plus ongoing rifampicin 300 mg daily. Amikacin was ceased due to ototoxicity but the rifampicin was well tolerated.

Within 4 weeks, a further nodule was found adjacent to the original right calf lesion and another nodule on the right buttock. With progression of both lesions over the following month despite the rifampicin therapy, a wide excision of the right buttock lesion with primary closure and clear histological margins, and a wide excision of the right calf lesion with a skin graft were performed. One of the histological margins of the calf lesion showed inflammation and necrosis. No tissue was sent for mycobacterial culture. She continued on rifampicin monotherapy.

An immunological screen with T-cell sub-sets, complement levels and immunoglobulin subclasses was normal, and a myeloma screen and HIV antibody test were negative.

Four weeks later, despite continuing rifampicin monotherapy, a swelling was noted on the dorsum of her left foot. This area was surgically debrided with macroscopic evidence of involvement of the base of the first metatarsal bone. A week later, she underwent further debridement and removal of a bony sequestrum from the first metatarsal. Histopathology of the bone revealed necrotising granulomatous inflammation with numerous AFB-positive organisms consistent with mycobacteria, however, mycobacterial cultures were not performed.

Over the following 2 months, she continued taking her rifampicin 300 mg daily with initial improvement in the foot, but then there was progression both clinically and on computed tomography examination. Therefore, ciprofloxacin 250 mg twice daily was added.

**Figure 1:**
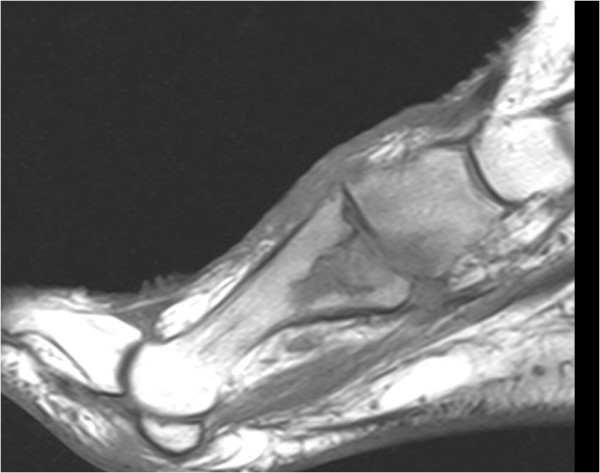
A magnetic resonance imaging scan of the foot showing the destructive changes of osteomyelitis in the proximal first metatarsal bone and adjacent medial cuneiform bone.

A magnetic resonance imaging scan performed a month later revealed ongoing destruction of the base and enhancement of the proximal two-thirds of the first metatarsal, with now the likely involvement of the first tarsometatarsal joint and the medial cuneiform bone (Figure 1). She was offered further extensive surgery to attempt removal of all infected bone but declined.

Remarkably, over the following 5 months, her wound and sinus healed and after 6 months of combined rifampicin and ciprofloxacin therapy, the antibiotics were ceased. Plain X-rays repeated after 6 months, and clinical follow-up for 36 months, revealed no evidence of persistent disease.

Sensitivity testing of her original isolate revealed it to be sensitive to rifampicin, ethambutol, amikacin, clarithromycin and ciprofloxacin.

## Discussion

This case demonstrates that successful treatment of *M. ulcerans *osteomyelitis and septic arthritis can be achieved with limited surgical debridement and 6 months of oral rifampicin and ciprofloxacin; this is, to the best of the authors' knowledge, the first time that this has been reported. Our patient's osteomyelitis progressed radiologically despite initial surgical debridement, but then resolved following the commencement of rifampicin and ciprofloxacin without further surgery, and with no local or distal recurrences in the 36-month follow-up period. Thus, we believe that this combination of antibiotics resulted in cure of the osteomyelitis in our case, and prevented the development of further metachronous *M. ulcerans *lesions. This raises the possibility that this simple and well-tolerated oral combination has the potential to reduce significantly the morbidity and disability that results worldwide in the surgical treatment of *M. ulcerans *osteomyelitis, and warrants further study.

*M. ulcerans *was first reported to cause osteomyelitis in 1993 [5], although recent reports suggest that it may complicate skin and subcutaneous disease in up to 13% of cases [1,6]. Usually *M. ulcerans *is thought to spread to bone contiguously from the skin, although metastatic spread via the blood stream or lymphatics has been proposed [5,6]. Owing to the lack of known effective medical treatment, surgery is usually required for cure and this often results in amputation or severe disability [1].

The support for the effectiveness of the antibiotics in our case is as follows. Firstly, *M. ulcerans *shows excellent in vitro sensitivity to rifampicin [7], and there is evidence from mouse models [8] and the treatment of early human BU lesions when combined with streptomycin [2], that rifampicin is also effective in vivo. Secondly, ciprofloxacin has good oral bio-availability and excellent penetration into bone and tissues, and *M. ulcerans *has also been shown to be highly susceptible to it in vitro [7]. Thirdly, the combination of rifampicin and ciprofloxacin may have acted synergistically in our patient as there is evidence in a mouse model of increased *M. ulcerans *bactericidal activity when rifampicin was combined with another fluoroquinolone antibiotic, sitafloxacin [9]. Finally, we have recently reported the benefit of adjunctive antibiotics in surgical treatment of BU, with especially promising results using the combination of rifampicin and ciprofloxacin [3].

There are anecdotal reports of spontaneous resolution of cutaneous *M. ulcerans *infection, but we are not aware of this occurring once osteomyelitis has developed. Nevertheless we cannot exclude this possibility. Furthermore, as rifampicin was used alone for 5 months prior to the commencement of ciprofloxacin, and with reports of rifampicin resistance developing when used as monotherapy [10], we cannot exclude the possibility that rifampicin resistance had developed in our isolate, and our patient responded to ciprofloxacin monotherapy. However, this in itself is worth noting.

We would normally recommend rifampicin at a dose of 10 mg/kg daily and ciprofloxacin 500 to 750 mg twice daily in healthy adults. However, lower doses were used in this case to improve tolerance in view of the patient's advanced age and frailty. Importantly, the regimen we prescribed allowed her to complete a 6-month course, compared with the treatment-limiting side effects she experienced from both clarithromycin and amikacin.

This case also illustrates several important points concerning the natural history and treatment of *M. ulcerans *infection. Firstly, *M. ulcerans *clearly has the potential to disseminate, via either the haematogenous or lymphatic route, resulting in multiple metachronous lesions, often in quite distant parts of the body. Our patient developed six lesions over 9 months, affecting both legs and the right buttock.

Secondly, although wide surgical excision is usually recommended as the mainstay of treatment [11], recurrences can occur both locally and distally. This is illustrated in our case with the development of five recurrent lesions, despite histology of the original excised lesion showing clear margins.

Thirdly, despite a known sensitivity of *M. ulcerans *to many drugs in vitro [7], the drugs have often not been effective in vivo [11]. In this case, despite the original isolate showing in vitro sensitivity to all the administered drugs, the patient continued to have recurrences whilst on combinations of rifampicin, clarithromycin and ethambutol; amikacin and rifampicin; and rifampicin alone. Although combinations of rifampicin and amikacin have shown good effectiveness in mice [8], in our case, this combination over 18 days failed to prevent further recurrences.

## Conclusion

*M. ulcerans *osteomyelitis can be successfully treated with limited surgical debridement and 6 months of oral rifampicin and ciprofloxacin. In addition, this antibiotic combination may prevent progression of human *M. ulcerans *disease. We believe that further studies evaluating this promising antibiotic combination with more conservative and less disabling surgery should be undertaken for both *M. ulcerans *osteomyelitis and *M. ulcerans *disease in general.

## Abbreviations

AFB: acid-fast bacilli; BU: Buruli or Bairnsdale ulcer; PCR: polymerase chain reaction.

## Competing interests

The authors declare that they have no competing interests.

## Authors' contributions

DPO conceived the study and drafted the manuscript. EA, AH, PDJ all helped to draft the manuscript. All authors read and approved the final manuscript.

## Consent

Written informed consent was obtained from the patient for publication of this case report and any accompanying images. A copy of the written consent is available for review by the Editor-in-Chief of this journal.
